# A Biomarker for Alzheimer’s Disease Based on Patterns of Regional Brain Atrophy

**DOI:** 10.3389/fpsyt.2019.00953

**Published:** 2020-01-14

**Authors:** Stefan Frenzel, Katharina Wittfeld, Mohamad Habes, Johanna Klinger-König, Robin Bülow, Henry Völzke, Hans Jörgen Grabe

**Affiliations:** ^1^ Department of Psychiatry and Psychotherapy, University Medicine Greifswald, Greifswald, Germany; ^2^ German Center for Neurodegenerative Diseases (DZNE), Greifswald, Germany; ^3^ Center for Biomedical Image Computing and Analytics, University of Pennsylvania, Philadelphia, PA, United States; ^4^ Institute of Diagnostic Radiology and Neuroradiology, University Medicine Greifswald, Greifswald, Germany; ^5^ Institute for Community Medicine, University Medicine Greifswald, Greifswald, Germany

**Keywords:** Alzheimer's disease, machine learning, dementia, magnetic resonance imaging, FreeSurfer

## Abstract

**Introduction:** It has been shown that Alzheimer’s disease (AD) is accompanied by marked structural brain changes that can be detected several years before clinical diagnosis *via* structural magnetic resonance (MR) imaging. In this study, we developed a structural MR-based biomarker for *in vivo* detection of AD using a supervised machine learning approach. Based on an individual’s pattern of brain atrophy a continuous AD score is assigned which measures the similarity with brain atrophy patterns seen in clinical cases of AD.

**Methods:** The underlying statistical model was trained with MR scans of patients and healthy controls from the Alzheimer’s Disease Neuroimaging Initiative (ADNI-1 screening). Validation was performed within ADNI-1 and in an independent patient sample from the Open Access Series of Imaging Studies (OASIS-1). In addition, our analyses included data from a large general population sample of the Study of Health in Pomerania (SHIP-Trend).

**Results:** Based on the proposed AD score we were able to differentiate patients from healthy controls in ADNI-1 and OASIS-1 with an accuracy of 89% (AUC = 95%) and 87% (AUC = 93%), respectively. Moreover, we found the AD score to be significantly associated with cognitive functioning as assessed by the Mini-Mental State Examination in the OASIS-1 sample after correcting for diagnosis, age, sex, age·sex, and total intracranial volume (Cohen’s f^2^ = 0.13). Additional analyses showed that the prediction accuracy of AD status based on both the AD score and the MMSE score is significantly higher than when using just one of them. In SHIP-Trend we found the AD score to be weakly but significantly associated with a test of verbal memory consisting of an immediate and a delayed word list recall (again after correcting for age, sex, age·sex, and total intracranial volume, Cohen’s f^2^ = 0.009). This association was mainly driven by the immediate recall performance.

**Discussion:** In summary, our proposed biomarker well differentiated between patients and healthy controls in an independent test sample. It was associated with measures of cognitive functioning both in a patient sample and a general population sample. Our approach might be useful for defining robust MR-based biomarkers for other neurodegenerative diseases, too.

## Introduction

Alzheimer’s disease (AD) is a neurodegenerative disorder and accounts for an estimated 60 to 80 percent of cases of dementia (1, 2). Dementia is characterized by memory impairments, disordered cognition, language problems, and changes in behaviour, which seriously impair a person’s ability to live independently. In advanced AD the person loses basic body functions like walking and swallowing and requires around the clock-care. According the World Health Organization (WHO) the incidence of dementia worldwide will reach about 135 million people in 2050 and will become a major challenge for health-care systems of western countries (3).

The hallmark pathology of AD is the progressive accumulation of amyloid beta protein and tau protein in the brain which is accompanied by death of neurons (1, 4). Macroscopically this is reflected in atrophy of specific brain regions which can be assessed *via* structural magnetic resonance (MR) imaging. At an early stage, the mild cognitive impairment phase, there typically is an atrophy only of the temporal lobe. With progression of the disease other cortical and subcortical regions, notably the hippocampus, become affected too (5–7). These structural changes have been shown to be detectable several years before the clinical diagnosis of AD (8, 9) which led to the development of imaging-based biomarkers of AD based on machine learning (10–16). Biomarkers based on structural MR imaging have been shown to differentiate well between cases of AD and cognitively healthy controls (17) and some of them have been shown to be sensitive at the preclinical stage (18). However, most of these biomarkers have been investigated in single cohorts only.

Since structural brain changes are detectable several years before clinical diagnosis MR-based biomarkers for AD are highly relevant for general population studies, too. However, the investigation of such biomarkers has gained attention only recently within the context of general brain ageing (19–21). In this study, we developed an MR-based biomarker for the *in vivo* assessment of AD based on a supervised machine learning approach. Based on an individual’s pattern of brain atrophy a continuous score is assigned which measures the similarity with brain atrophy patterns seen in clinical cases of AD. The underlying statistical model is trained using data from the Alzheimer’s Disease Neuroimaging Initiative (ADNI) (22) and validation is performed in an independent patient sample from the Open Access Series of Imaging Studies (OASIS) (23). Finally, our proposed biomarker is investigated in general population data from the Study of Health in Pomerania (SHIP-Trend) (24).

## Materials and Methods

### Sample Description

#### Alzheimer’s Disease Neuroimaging Initiative (ADNI)

Data used in the preparation of this article were obtained from the Alzheimer’s Disease Neuroimaging Initiative (ADNI) database (adni.loni.usc.edu). The ADNI was launched in 2003 as a public-private partnership, led by Principal Investigator Michael W. Weiner, MD. The primary goal of ADNI has been to test whether serial magnetic resonance (MR) imaging, positron emission tomography (PET), other biological markers, and clinical and neuropsychological assessment can be combined to measure the progression of mild cognitive impairment (MCI) and early Alzheimer’s disease (AD). For up-to-date information, see www.adni-info.org. T1-weighted structural MR scans from 413 participants of the ADNI-1 screening sample were considered in this study. Images were acquired using multiple scanners with a field strength of 1.5T (25, 26). The detailed MR protocol can be found in the supplement. Since the ADNI scans were used to train the AD classifier additional quality control of the image processing was performed as explained below. The final sample comprised N = 374 individuals with 165 diagnosed with AD and 209 cognitively healthy controls (CN) (see **Table 1**).

**Table 1:** Basic demographic characteristics of all three samples.

	ADNI-1 screening	OASIS-1	SHIP-Trend
N	374	416	1,973
Females	186 (49%)	254 (61%)	1,038 (53%)
AD	165 (44%)	100 (24%)	–
Age [y]	75.7 (6.3)	52.9 (25.0)	51.3 (14.0)
Intracranial Volume [dl]	15.4 (1.7)	14.8 (1.6)	15.9 (1.6)

#### Open Access Series of Imaging Studies (OASIS)

To validate the AD classifier we used data from the Open Access Series of Imaging Studies (OASIS-1) which is a cross-sectional collection of MR scans of N = 416 individuals aged 18 to 96 (23) (see **Table 1**). One hundred of the participants older than 60 have been clinically diagnosed with very mild to moderate AD. More information can be found at www.oasis-brains.org. Details of the MR protocol can be found in the supplement. All images were screened for artefacts, acquisition problems, and processing errors and images with severe flaws were excluded by the OASIS investigators. No additional quality control was performed by the authors. 235 participants (100 AD, 135 CN) completed the Mini-Mental State Examination (MMSE). The MMSE is a 30-point questionnaire that is used extensively to screen for dementia (27).

#### Study of Health in Pomerania (SHIP-Trend)

The Study of Health in Pomerania (SHIP) was designed to assess the prevalence of common risk factors and diseases in a population of northeast Germany randomly drawn from local registers (24). 4,308 subjects participated at baseline between 1997 and 2001. In parallel to the original SHIP study a new independent sample was drawn and examinations of similar extent were undertaken (SHIP-Trend). In this study, T1-weighted structural MR images of the head from 2,154 participants of SHIP-Trend were considered (28). Details of the MR protocol can be found in the supplement. Scans with very poor technical quality, (e.g. frontal darkening) were excluded (N = 84). In addition, scans showing structural abnormalities (e.g. tumors, cysts) and cases of cerebral stroke were excluded as well (N = 93). The image processing pipeline (see below) failed to process 4 scans. The final sample comprised N = 1973 individuals (see **Table 1**).

Of those, 1,955 participants completed a word list recall (WLR) test during the face-to-face interview as part of the standard SHIP-Trend protocol. The WLR test consists of eight items which needed to be recalled immediately (immediate WLR, 0 to 8 points) and after a 20 min delay (delayed WLR with distractor words, -8 to 8 points). The total WLR score was computed as sum of both tests. The WLR is part of the Nuremberg Gerontopsychological Inventory (29).

### MR Image Segmentation With Freesurfer

Cortical reconstruction and volumetric segmentation of all three data sets were performed with the FreeSurfer image analysis suite version 5.3 (“recon-all”), which is documented and freely available for download online (http://surfer.nmr.mgh.harvard.edu).

Briefly, this processing includes removal of non-brain tissue using a hybrid watershed/surface deformation procedure (30), automated Talairach transformation, segmentation of subcortical white matter and deep gray matter volumetric structures (including hippocampus, amygdala, caudate, putamen, ventricles) (31–33), intensity normalization (34), tessellation of the gray matter white matter boundary, automated topology correction (35, 36), and surface deformation following intensity gradients to optimally place the gray/white and gray/cerebrospinal fluid borders at the location where the greatest shift in intensity defines the transition to the other tissue class (37–39).

Once the cortical models are complete, individual images are being registered to a spherical atlas which is based on individual cortical folding patterns to match cortical geometry across subjects (40), and the cerebral cortex is being parcelled into 68 units with respect to gyral and sulcal structure (41, 42). Cortical white matter, i.e. white matter up to 5mm below the gray matter boundary, is also being parcelled into 68 units by assigning each white matter voxel the label of the closest cortical voxel (43). FreeSurfer also gives an estimate of the total intracranial volume (eTIV) which was not used to train the AD classifier but as a covariate in subsequent statistical analyses.

**Figure 1:**
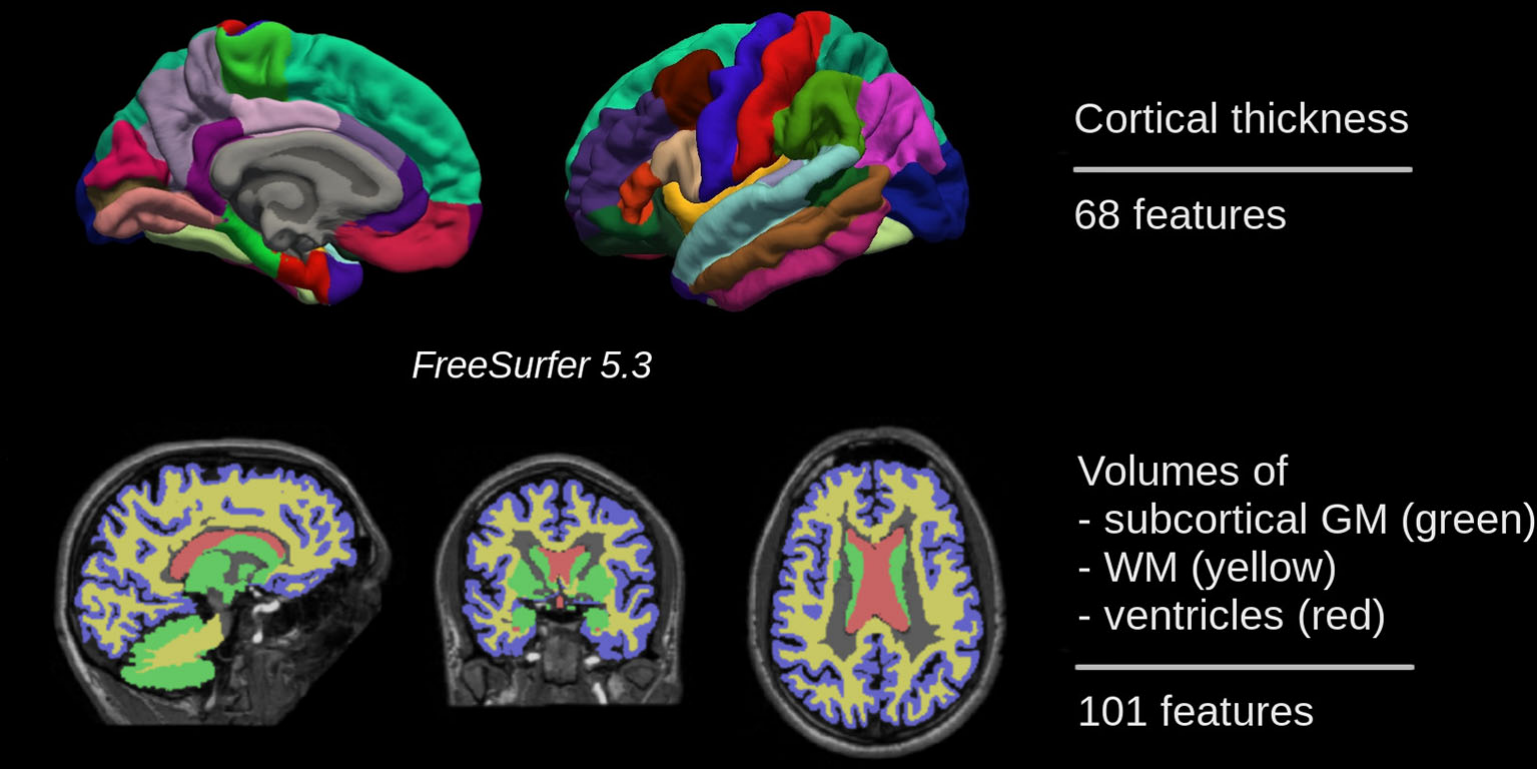
In total, 169 features of gray matter, white matter, and the ventricular system were used for training a binary classifier which distinguishes between individuals with Alzheimer’s disease and cognitively normal ones.

Although being part of the standard FreeSurfer output several brain regions were excluded from the analyses. The 5^th^ ventricle was excluded because it was not detected in all scan (zero volume). In addition, the brain stem and optic chiasm were excluded as well. In total, 169 out of 172 brain regions of gray matter, white matter, and the ventricular system were considered (see **Figure 1**). The complete list of regions can be found in the **Supplementary Material**.

### Alzheimer’s Disease Classifier

Based on the ADNI-1 screening sample a binary classifier was trained with diagnoses as dependent variable. In order to minimize the influence of image segmentation errors on the classifier, we performed an additional statistical quality control of each feature. More specifically, we removed all scans with brain measurements deviating more than four standard deviations from the mean value after adjusting for age, sex, age·sex, eTIV, and diagnosis (N = 39). All features were standardized to zero mean and unit variance. We then used L2-penalized (ridge) logistic regression to train the binary classifier which optimally separates individuals with AD from CN (44). The AD score was defined as the linear predictors of the logistic model, i.e. it is given by log[p/(1-p)] with p denoting the probability of having AD.

Prediction of AD scores in OASIS-1 and SHIP-Trend were based on a classifier trained on the whole ADNI-1 sample. The corresponding model coefficients can be found in the supplement. The penalization parameter λ was selected from the set {2^-8^, 2^-7^, …, 2} by 20-fold cross-validation with 20 repetitions (λ = 0.125) anduni-modality of the tuning curve was checked by visual inspection (see **Supplementary Material**). In order to assess the classification accuracy within ADNI-1 we used leave-one-out cross-validation, i.e. each individual’s AD score was calculated using a model trained on all others. The optimal λ was estimated within a second loop in order to strictly separate training and test data (again by 20-fold cross-validation with 20 repetitions).

### Voxel-Based Morphometry

For SHIP-Trend we additionally performed voxel-based morphometry (VBM) analyses with SPM12 (Welcome Trust Centre for Neuroimaging, University College London) and CAT12 [developed by Christian Gaser, University of Jena, Germany, http://www.neuro.uni-jena.de, e.g. (45)] in order to map the contribution of distinct brain regions to the AD score.

All images were bias-corrected, spatially normalized by using the high-dimensional DARTEL normalization, segmented into the different tissue classes, modulated for non-linear warping and affine transformations, and smoothed by a Gaussian kernel of 8 mm FWHM. The homogeneity of gray matter images was checked using the covariance structure of each image with all other images (outliers ≥3 standard deviations from the mean), as implemented in the check data quality function in the CAT12 toolbox. To mask irrelevant brain areas of the smoothed gray and white matter segmentations we used the Masking Toolbox from Gerard Ridgway to define explicit masks for the gray and white matter VBM analyses. Specifically, we used the MATLAB script “make_majority_mask.m” to generate a gray matter mask with an absolute threshold of 0.1 and a consensus fraction of 80% and a white matter mask with an absolute threshold of 0.2 and a consensus fraction of 90%.

The statistical threshold for significant voxels was set to a family-wise error (FWE) corrected peak-level p-values P_peak,FWE_ < 0.025 as we conducted a two-sided test and looked at positive and negative associations with the FSAD score while correcting for age, sex, age·sex, and total intracranial volume. Again, age was modeled by restricted cubic splines with four knots located at the 0.05, 0.33, 0.66, and 0.95 age quantiles.

### Statistical Analysis

All statistical analyses were performed with R 3.6 (46). The classifier was implemented using the *glmnet* package (47). Association analyses of the AD score with the basic covariates age, sex, age·sex, eTIV, and diagnosis were performed by ordinary least-squares multivariable regression. For SHIP-Trend we used restricted cubic splines (48) with four knots located at the 0.05, 0.33, 0.66, and 0.95 quantile in order to account for the non-linear dependency of the AD score on chronological age. Effects of single variables were assessed either by t-tests with robust variance estimates or ANOVA of type 2.

## Results

Prior to training the AD classifier we checked the ADNI-1 screening sample for possible imbalances with respect to age, sex, and intracranial volume. We did not find significant differences between patients and controls with respect to age (t = -0.55, P = 0.58), sex (Fisher’s Exact Test, P = 0.84), and estimated intracranial volume (t = 0.15, P = 0.88).

### Prediction of Diagnoses in ADNI-1 and OASIS-1 Based on the AD Score

**Figure 2:**
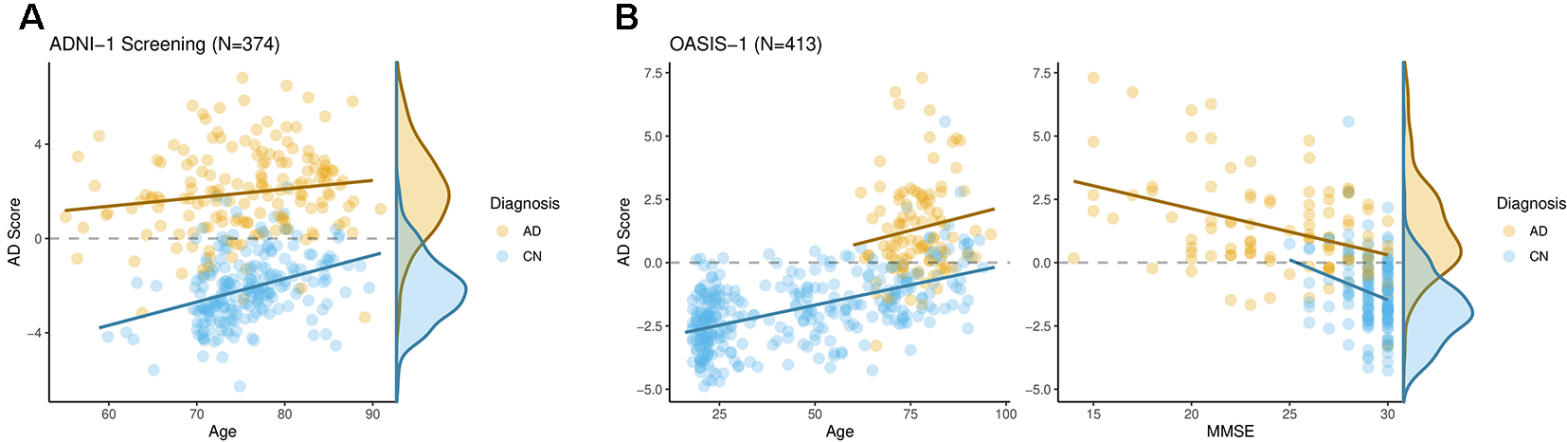
The AD score differentiated well individuals with Alzheimer’s disease from cognitively normal ones both in ADNI-1 **(A)** and OASIS-1 **(B)**. Moreover, it was significantly associated with cognitive functioning as assessed by the Mini-Mental State Examination within both groups in OASIS-1.

At first, classification performance within the ADNI-1 screening sample was investigated. Classification accuracy was assessed by leave-one-out cross-validation, i.e. each individual’s AD score was calculated using a model trained on all others. The resulting scores are shown in **Figure 2A**. Individuals with an AD score larger than zero and smaller than zero were classified as AD and CN, respectively, and these classifications were compared with the known diagnoses. The overall accuracy was 89% with the 95% confidence interval (CI) (85.7%, 92.2%). Sensitivity (true positive rate) and specificity (true negative rate) was 91% and 87%, respectively. The receiver operating characteristic (ROC) curves were obtained by systematic variation of the classification threshold and area under the curve (AUC) was calculated as 95% with 95% CI (93.5%, 97.6%).

Using the ADNI-1 sample a model was trained and AD scores were calculated for the OASIS-1 sample. The resulting scores are shown in **Figure 2B**, left panel. Again, individuals with an AD score larger than zero and smaller than zero were classified as AD and CN, respectively. The overall accuracy was 87% with 95% CI (83.2%, 90.0%). Sensitivity and specificity were 89% and 79%, respectively. The AUC was calculated as 93% with 95% CI (90.0%, 95.7%).

### Association Analyses in ADNI-1 and OASIS-1

We performed association analyses of the AD score with the basic covariates diagnosis, age, sex, age·sex, and intracranial volume by means of multivariable regression. For the ADNI-1 sample the percentage of variation explained (R^2^) was 72%. As expected, the AD score was significantly larger in those diagnosed with AD (t = 30, P < 2·10^-16^, see **Figure 2A**). In addition, there was a significant effect of age (t = 2.5, P = 0.012). No significant effects of sex (t = 1.1, P = 0.29), age·sex (t = -0.89, P = 0.37), or intracranial volume (t = 1.4, P = 0.17) were found.

For the OASIS-1 sample the multivariable regression of the AD score yielded R^2^^=^ 55%. Again, we found a significant effect of diagnosis of AD (t = 9.7, P < 2·10^-16^), and age (t = 8.5, P = 4.9·10^-16^). In addition, there was a significant effect of sex with females having slightly larger AD scores (t = 2.2, P = 0.025). No significant effects of age·sex (t = -0.75, P = 0.45), or intracranial volume (t = 0.55, P = 0.57) were found.

When analyzing the OASIS-1 subsample with MMSE scores available (N = 235, 100 AD, 135 CN) we again found significant effects of diagnosis (t = 9.3, P < 2.2·10^-16^), and age (t = 5.7, P = 3.9·10^-8^). No significant effects were found for sex (t = 0.97, P = 0.33), age·sex (t = -0.61, P = 0.54), and intracranial volume (t = 0.80, P = 0.42). The total R^2^ was 45%. Adding the MMSE score to the model increased the R^2^ to 51% and the corresponding marginal effect was significant (t = -4.1, P = 4.9·10^-5^, Cohen’s f^2^ = 0.13), i.e. on average individuals with low MMSE scores had larger AD scores when correcting for all basic covariates including diagnosis.

### Prediction of Diagnoses Using Both the AD Score and the MMSE Score in OASIS-1

In order to compare the diagnostic utility of the AD score with the MMSE we aimed to predict diagnoses in the OASIS-1 subsample with MMSE scores available. For this we used standard logistic regression models with different sets of predictors and compared the corresponding classification accuracies. Note that we did not separate the training and test set since we aimed to compare different sets of predictors rather than obtaining objective accuracy estimates. Using a basic model containing age, sex, and its interaction, we were able to predict AD diagnoses with an accuracy of 61% (AUC = 70%). Adding either the MMSE score or the AD score improved the accuracy to 82% (AUC = 91%) and 82% (AUC = 90%), respectively. When adding both the MMSE score and the AD score the resulting accuracy improved even further to 87% (AUC = 94%). The accuracy of the combined model was significantly better than one of the two previous ones (χ_1_^2^ = 29, P = 8·10^-8^; χ_1_^2^^=^ 53, P = 3·10^-13^).

### General Population Data From the SHIP Sample

**Figure 3:**
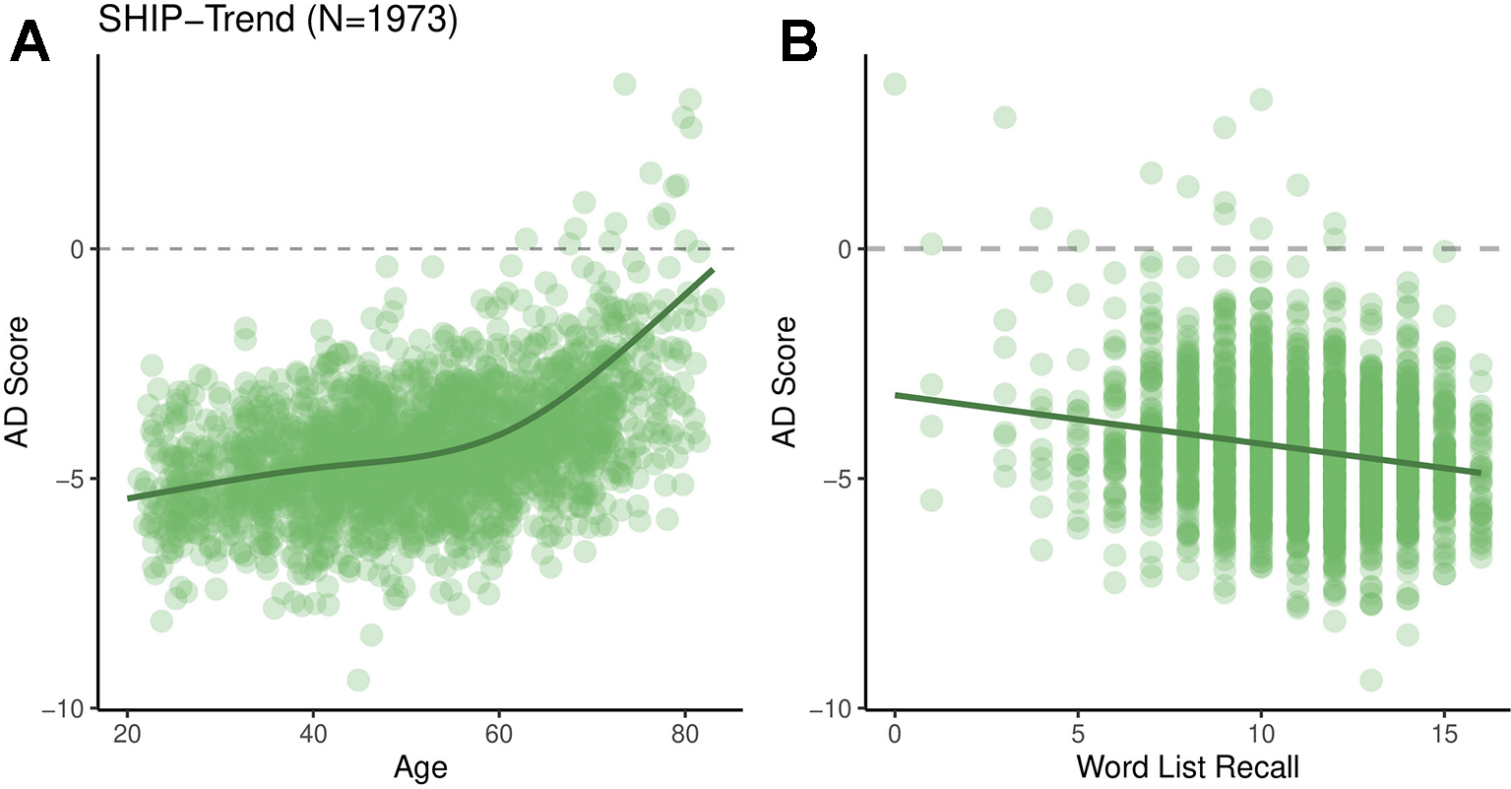
AD scores for the SHIP-Trend sample plotted against chronological age **(A)** and the overall word list recall score **(B)**.

AD scores were calculated for the SHIP-Trend sample (N = 1973, see **Table 1**) using a model trained on the whole ADNI-1 screening sample. Again, we performed association analyses of the AD score with the basic covariates age, sex, age·sex, and intracranial volume by means of multivariable regression. Since the AD score was clearly non-linearly related to age (see **Figure 3A**) we decided to include age by restricted cubic splines. ANOVA of type 2 was used to assess the effects of each variable. We found significant associations with age (F = 170, P < 2·10^-16^) and age·sex (F = 3.7, P = 0.010). No significant effects of sex (F = 0.40, P = 0.53), or intracranial volume (t = 2.5, P = 0.11) were found. The R^2^ was 22%.

The AD score was significantly associated with the total WLR score (F = 4.1, P = 0.037, Cohen’s f^2^^=^ 0.009, adjusted for all basic covariates, see **Figure 3B**). Additional analyses showed that the AD score was more strongly associated with the immediate WLR score (F = 4.9, P = 0.026) than the delayed WLR recall (F = 1.8, P = 0.17).

**Figure 4:**
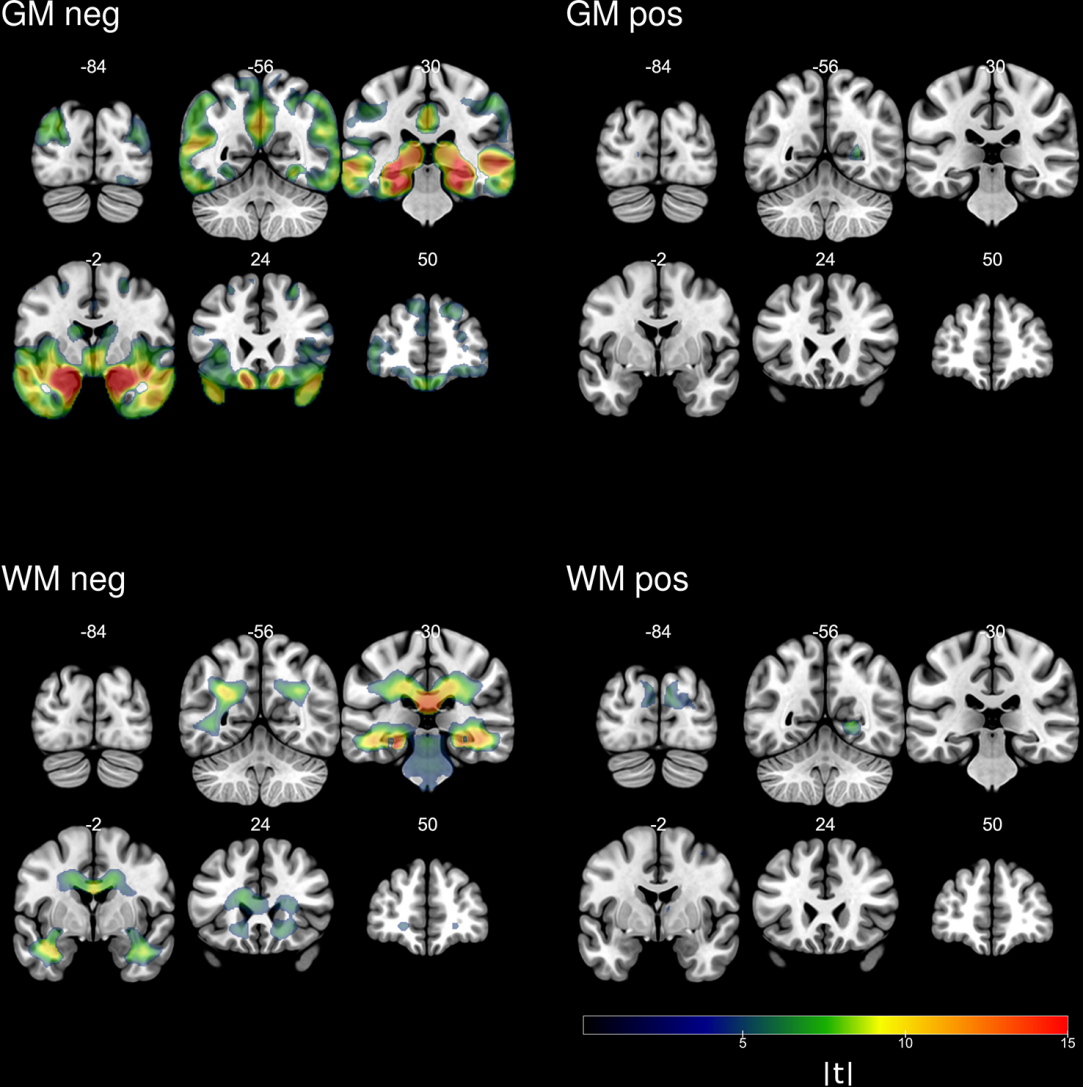
Absolute values of the t-statistics of voxel-based analyses of the AD score in SHIP-Trend.

In order to map the contributions of distinct brain regions to the AD score in greater detail we performed VBM analyses with both gray and white matter segmentations in SHIP-Trend. The results are visualized in **Figure 4**. Using the gray matter segmentation we found a large cluster that was negatively associated with the AD score. The peak voxel was located in the left medial temporal gyrus. The cluster stretched over the medial temporal gyrus, the inferior temporal gyrus, the fusiform gyrus, and the precuneus in both hemispheres, among others. Using the white matter segmentation we also found a large cluster that was negatively associated with the AD score. It comprised the medial temporal lobe, the periventricular area, and the corpus callosum, among others. Interestingly, it also includes a large portion of the brain stem which was not included in the feature set used for constructing the AD score.

## Discussion

In this study, we developed a structural MR imaging-based biomarker for the *in vivo* detection of Alzheimer’s disease. It was based on 169 regional brain features of gray matter, white matter, and the ventricular system derived from the image processing pipeline FreeSurfer. L2-penalized logistic regression was used to define a binary classifier which optimally separates individuals with AD from cognitively normal ones. For the ADNI-1 screening sample the cross-validated classification accuracy was 89% and AUC was 95%. These results are on par with other classification studies involving structural MR images (17). However, most classification studies were based on only one sample. Here, the classifier was trained using the ADNI-1 screening sample and AD scores were predicted in the independent sample OASIS-1. We found our classifier to also perform well with an accuracy of 87% and AUC being 93%.

For obtaining regional brain features we used the freely available image segmentation pipeline FreeSurfer. FreeSurfer has been shown to give reliable volumetric estimates independent of scanner platforms and protocols with the exception of the magnetic field strength which has been found to introduce additional bias (49). In our study, however, all scans were acquired with 1.5T. Since FreeSurfer is available under an open source license for the GNU/Linux operating system it can be run within typical high performance computing environments with little to no additional adaptations. This facilitates the application to large imaging data sets which are being used increasingly for the investigation of neurodegenerative disorders. Moreover, future improvements of the image processing algorithms used within FreeSurfer will likely improve any derived biomarkers, too.

On the other hand there is strong evidence for at least three distinct subtypes of AD with respect to regional brain atrophy (50, 51). Hence, it is unclear whether further improvements of the classification accuracy of structural MRI markers with respect a single diagnostic category (AD diagnosis) can be expected. Instead, the relation of MRI markers measures and measures of cognitive functioning, which ultimately impairs the affected individual’s quality of life, seems to be more appropriate. Here, we studied the association of the AD score with MMSE scores in a subsample of OASIS-1. We found a significant association after correcting for diagnosis, age, sex, age·sex, and total intracranial volume (Cohen’s f^2^ = 0.13, see **Figure 2B**). The AD score was associated with cognitive functioning in AD patients (adjusted for age, sex, and intracranial volume) which indicates it to be a measure of the progression of AD. Interestingly, it was also associated with the MMSE in cognitive normal individuals after correcting for age, sex, and intracranial volume, indicating that it captures subclinical pathology (atrophy), too.

This was supported by the association analyses in the general population sample SHIP-Trend where we found the AD score to be significantly associated with the WLR consisting of an immediate and a delayed recall (again after correcting for age, sex, age·sex, and total intracranial volume, Cohen’s f^2^ = 0.009). This association was mainly driven by the immediate recall. Indeed, there seems to be a deficit in semantic memory years before AD diagnosis while AD patients show impairments in multiple cognitive domains (52). Such a deficit in semantic memory could explain the association with the WLR performance in SHIP-Trend.

However, the association between the AD score and cognitive functioning in non-demented individuals could also be partially driven by other psychiatric diseases. One example for this is depression which is known to be associated with decreased hippocampal volume and impaired memory. Since depression has a much higher life-time prevalence than AD it is potentially highly relevant for population-based studies. Whether the AD score proposed here is indeed associated with a specific profile of cognitive dysfunction in non-demented individuals needs to be investigated in future studies.

One limitation of our method is that AD scores of single individuals can only be interpreted within populations after adjusting for confounding variables like age. In all data sets the AD score was positively associated with age. In SHIP-Trend this association was non-linear with the slope increasing around the age of 60 (see **Figure 3A**). However, this should not be interpreted as progression of some sort of AD-related subclinical pathology, but rather statistical artefact of the spatial overlap of general age-related atrophy and AD-related atrophy. Even if the model coefficients of the AD classifier were randomly drawn there would still be a significant association of the resulting AD score with chronological age. Since age is a potential confounding variable thorough adjustment of the analyses is needed. Most of the time this requires non-linear modelling with polynomials or splines.

In summary, our proposed AD score well differentiated between patients and healthy controls in an independent test sample. It was associated with measures of cognitive functioning both in a patient sample and a general population sample. Thus, our approach might be useful for defining robust MR-based biomarkers for other neurodegenerative diseases, too.

## Data Availability Statement

Data used in the preparation of this article were obtained from the Alzheimer’s Disease Neuroimaging Initiative (ADNI) database (adni.loni.usc.edu) and the Open Access Series of Imaging Studies. Request should be made to the corresponding author: stefan.frenzel@uni-greifswald.de.

## Ethics Statement

The studies involving human participants were reviewed and approved by Institutional Review Board of University Medicine Greifswald (“Ethikkomission an der Universitätsmedizin Greifswald”). The patients/participants provided their written informed consent to participate in this study. Written informed consent was obtained from the individual(s) for the publication of any potentially identifiable images or data included in this article.

## Author Contributions

SF performed all statistical analysis, and wrote the manuscript. SF, JK-K, MH, and HG designed the study. SF, MH, and KW processed the MR imaging data. KW conducted the VBM analyses. RB and HV contributed essentially to the data collection.

## Conflict of Interest

HG has received travel grants and speakers honoraria from Fresenius Medical Care and Janssen Cilag. He has received research funding from the German Research Foundation (DFG), the German Ministry of Education and Research (BMBF), the DAMP Foundation, Fresenius Medical Care, the EU “Joint Programme Neurodegenerative Disorders (JPND)”.

The remaining authors declare that the research was conducted in the absence of any commercial or financial relationships that could be construed as a potential conflict of interest.

The reviewer XH declared a shared affiliation, with no collaboration, with one of the authors MH to the handling editor.

## References

[1] Alzheimer’s Association 2016 Alzheimer’s disease facts and figures. Alzheimers Dement (2016) 12:459–509. 10.1016/j.jalz.2016.03.001 27570871

[2] Barker WW Luis CA Kashuba A Luis M Harwood DG Loewenstein D Relative frequencies of alzheimer disease, lewy body, vascular and frontotemporal dementia, and hippocampal sclerosis in the State of Florida brain bank. Alzheimer Dis Assoc Disord (2002) 16:203. 10.1097/00002093-200210000-00001 12468894

[3] Prince MJ Guerchet MM Prina M The Epidemiology and Impact of Dementia: Current State and Future Trends. Geneva, Switzerland: WHO Thematic Briefing (2015).

[4] Hardy JA Higgins GA Alzheimer’s disease: the amyloid cascade hypothesis. Science (1992) 256(5054):184–5. 10.1126/science.1566067 1566067

[5] Frisoni GB Fox NC Jack CR Jr. Scheltens P Thompson PM The clinical use of structural MRI in Alzheimer disease. Nat Rev Neurol (2010) 6:67–77. 10.1038/nrneurol.2009.215 20139996 PMC2938772

[6] Thompson PM Hayashi KM Zubicaray G de, Janke AL Rose SE Semple J Dynamics of gray matter loss in Alzheimer’s Disease. J Neurosci (2003) 23:994–1005. 10.1523/JNEUROSCI.23-03-00994.2003 12574429 PMC6741905

[7] Tondelli M Wilcock GK Nichelli P De Jager CA Jenkinson M Zamboni G Structural MRI changes detectable up to ten years before clinical Alzheimer’s disease. Neurobiol Aging (2012) 33, 825:e25–825.e36. 10.1016/j.neurobiolaging.2011.05.018 21782287

[8] Jack CR Shiung MM Weigand SD O’Brien PC Gunter JL Boeve BF Brain atrophy rates predict subsequent clinical conversion in normal elderly and amnestic MCI. Neurology (2005) 65:1227–31. 10.1212/01.wnl.0000180958.22678.91 PMC2753547 16247049

[9] Twamley EW Ropacki SAL Bondi MW Neuropsychological and neuroimaging changes in preclinical Alzheimer’s disease. J Int Neuropsychol Soc (2006) 12:707–35. 10.1017/S1355617706060863 PMC1621044 16961952

[10] Beheshti I Demirel H Feature-ranking-based Alzheimer’s disease classification from structural MRI. Magnet Resonance Imaging (2016) 34:252–63. 10.1016/j.mri.2015.11.009 26657976

[11] Davatzikos C Xu F An Y Fan Y Resnick SM Longitudinal progression of Alzheimer’s-like patterns of atrophy in normal older adults: the SPARE-AD index. Brain (2009) 132:2026–35. 10.1093/brain/awp091 PMC2714059 19416949

[12] Duchesne S Caroli A Geroldi C Barillot C Frisoni GB Collins DL MRI-based automated computer classification of probable AD versus normal controls. IEEE Trans Med Imaging (2008) 27:509–20. 10.1109/TMI.2007.908685 18390347

[13] Fan Y Resnick SM Wu X Davatzikos C Structural and functional biomarkers of prodromal Alzheimer’s disease: a high-dimensional pattern classification study. NeuroImage (2008) 41:277–85. 10.1016/j.neuroimage.2008.02.043 PMC2682533 18400519

[14] Klöppel S Stonnington CM Chu C Draganski B Scahill RI Rohrer JD Automatic classification of MR scans in Alzheimer’s disease. Brain (2008) 131:681–9. 10.1093/brain/awm319 PMC2579744 18202106

[15] Li H Habes M Wolk DA Fan Y Alzheimer’s Disease Neuroimaging Initiative and the Australian Imaging Biomarkers and Lifestyle Study of Aging A deep learning model for early prediction of Alzheimer’s disease dementia based on hippocampal magnetic resonance imaging data. Alzheimers Dement (2019) 15:1059–70. 10.1016/j.jalz.2019.02.007 PMC6719787 31201098

[16] Salvatore C Cerasa A Battista P Gilardi MC Quattrone A Castiglioni I Magnetic resonance imaging biomarkers for the early diagnosis of Alzheimer’s disease: a machine learning approach. Front Neurosci (2015) 9:307. 26388719 10.3389/fnins.2015.00307 PMC4555016

[17] Rathore S Habes M Iftikhar MA Shacklett A Davatzikos C A review on neuroimaging-based classification studies and associated feature extraction methods for Alzheimer’s disease and its prodromal stages. NeuroImage (2017) 155:530–48. 10.1016/j.neuroimage.2017.03.057 PMC5511557 28414186

[18] Davatzikos C Bhatt P Shaw LM Batmanghelich KN Trojanowski JQ Prediction of MCI to AD conversion, via MRI, CSF biomarkers, and pattern classification. Neurobiol Aging (2011) 32, 2322:e19–27. 10.1016/j.neurobiolaging.2010.05.023 PMC2951483 20594615

[19] Cole JH Ritchie SJ Bastin ME Valdés Hernández MC Muñoz Maniega S Royle N Brain age predicts mortality. Mol Psychiatry (2018) 23:1385–92. 10.1038/mp.2017.62 PMC5984097 28439103

[20] Habes M Janowitz D Erus G Toledo JB Resnick SM Doshi J Advanced brain aging: relationship with epidemiologic and genetic risk factors, and overlap with Alzheimer disease atrophy patterns. Trans Psychiatry (2016) 6:e775. 10.1038/tp.2016.39 PMC4872397 27045845

[21] Janowitz D Habes M Toledo JB Hannemann A Frenzel S Terock J Inflammatory markers and imaging patterns of advanced brain aging in the general population. Brain Imaging Behav (2019). 10.1007/s11682-019-00058-y PMC8374834 30820858

[22] Petersen RC Aisen PS Beckett LA Donohue MC Gamst AC Harvey DJ Alzheimer’s Disease Neuroimaging Initiative (ADNI). Neurology (2010) 74:201–9. 10.1212/WNL.0b013e3181cb3e25 PMC2809036 20042704

[23] Marcus DS Wang TH Parker J Csernansky JG Morris JC Buckner RL Open Access Series of Imaging Studies (OASIS): cross-sectional MRI data in young, middle aged, nondemented, and demented older adults. J Cognit Neurosci (2007) 19:1498–507. 10.1162/jocn.2007.19.9.1498 17714011

[24] Völzke H Alte D Schmidt CO Radke D Lorbeer R Friedrich N Cohort profile: the study of health in Pomerania. Int J Epidemiol (2011) 40:294–307. 20167617 10.1093/ije/dyp394

[25] Jack CR Bernstein MA Fox NC Thompson P Alexander G Harvey D The Alzheimer’s disease neuroimaging initiative (ADNI): MRI methods. J Magnet Resonance Imaging (2008) 27:685–91. 10.1002/jmri.21049 PMC2544629 18302232

[26] Wyman BT Harvey DJ Crawford K Bernstein MA Carmichael O Cole PE Standardization of analysis sets for reporting results from ADNI MRI data. Alzheimer’s Dementia: J Alzheimer’s Assoc (2013) 9:332–7. 10.1016/j.jalz.2012.06.004 PMC3891834 23110865

[27] Folstein MF Folstein SE McHugh PR “Mini-mental state”: a practical method for grading the cognitive state of patients for the clinician. J Psychiatr Res (1975) 12:189–98. 10.1016/0022-3956(75)90026-6 1202204

[28] Hegenscheid K Kühn JP Völzke H Biffar R Hosten N Puls R Whole-body magnetic resonance imaging of healthy volunteers: pilot study results from the population-based SHIP study. Rofo (2009) 181:748–59. 10.1055/s-0028-1109510 19598074

[29] Oswald WD Fleischmann UM Psychometrics in aging and dementia: advances in geropsychological assessments. Arch Gerontol Geriatr (1985) 4:299–309. 3833084 10.1016/0167-4943(85)90037-8

[30] Ségonne F Dale AM Busa E Glessner M Salat D Hahn HK A hybrid approach to the skull stripping problem in MRI. Neuroimage (2004) 22:1060–75. 10.1016/j.neuroimage.2004.03.032 15219578

[31] Fischl B Salat DH Busa E Albert M Dieterich M Haselgrove C Whole brain segmentation: automated labeling of neuroanatomical structures in the human brain. Neuron (2002) 33:341–55. 10.1016/s0896-6273(02)00569-x 11832223

[32] Fischl B Salat DH van der Kouwe AJW Makris N Ségonne F Quinn BT Sequence-independent segmentation of magnetic resonance images. Neuroimage (2004a) 23 Suppl 1:S69–84. 10.1016/j.neuroimage.2004.07.016 15501102

[33] Han X Fischl B Atlas renormalization for improved brain MR image segmentation across scanner platforms. IEEE Trans Med Imaging (2007) 26:479–86. 10.1109/TMI.2007.893282 17427735

[34] Sled JG Zijdenbos AP Evans AC A nonparametric method for automatic correction of intensity nonuniformity in MRI data. IEEE Trans Med Imaging (1998) 17:87–97. 9617910 10.1109/42.668698

[35] Fischl B Liu A Dale AM Automated manifold surgery: constructing geometrically accurate and topologically correct models of the human cerebral cortex. IEEE Trans Med Imaging (2001) 20:70–80. 11293693 10.1109/42.906426

[36] Ségonne F Pacheco J Fischl B Geometrically accurate topology-correction of cortical surfaces using nonseparating loops. IEEE Trans Med Imaging (2007) 26:518–29. 10.1109/TMI.2006.887364 17427739

[37] Dale AM Sereno MI Improved localizadon of cortical activity by combining EEG and MEG with MRI cortical surface reconstruction: a linear approach. J Cognit Neurosci (1993) 5:162–76. 10.1162/jocn.1993.5.2.162 23972151

[38] Dale AM Fischl B Sereno MI Cortical surface-based analysis. I. Segmentation and surface reconstruction. Neuroimage (1999) 9:179–94. 10.1006/nimg.1998.0395 9931268

[39] Fischl B Dale AM Measuring the thickness of the human cerebral cortex from magnetic resonance images. Proc Natl Acad Sci USA (2000) 97:11050–5. 10.1073/pnas.200033797 PMC27146 10984517

[40] Fischl B Sereno MI Tootell RBH Dale AM High-resolution intersubject averaging and a coordinate system for the cortical surface. Hum Brain Mapp (1999) 8:272–84. 10.1002/(SICI)1097-0193(1999)8:4<272::AID-HBM10>3.0.CO;2-4 PMC6873338 10619420

[41] Desikan RS Ségonne F Fischl B Quinn BT Dickerson BC Blacker D An automated labeling system for subdividing the human cerebral cortex on MRI scans into gyral based regions of interest. Neuroimage (2006) 31:968–80. 10.1016/j.neuroimage.2006.01.021 16530430

[42] Fischl B van der Kouwe A Destrieux C Halgren E Ségonne F Salat DH Automatically parcellating the human cerebral cortex. Cereb Cortex (2004b) 14:11–22. 14654453 10.1093/cercor/bhg087

[43] Salat DH Greve DN Pacheco JL Quinn BT Helmer KG Buckner RL Regional white matter volume differences in nondemented aging and Alzheimer’s disease. Neuroimage (2009) 44:1247–58. 10.1016/j.neuroimage.2008.10.030 PMC2810540 19027860

[44] Hastie T Tibshirani R Friedman JH The Elements of Statistical Learning: Data Mining, Inference, and Prediction. Berlin, Germany: Springer Science & Business Media (2001).

[45] Penny WD Friston KJ Ashburner JT Kiebel SJ Nichols TE Statistical Parametric Mapping: The Analysis of Functional Brain Images. Amsterdam, Netherlands: Elsevier (2011).

[46] Team T (2008). The R project for statistical computing. http://www.r-Project.Org.

[47] Friedman J Hastie T Tibshirani R Regularization paths for generalized linear models via coordinate descent. J Stat Softw (2010) 33:1–22. 10.18637/jss.v033.i01 20808728 PMC2929880

[48] Harrell F Regression Modeling Strategies: With Applications to Linear Models, Logistic and Ordinal Regression, and Survival Analysis. Basel, Switzerland: Springer International Publishing (2015).

[49] Han X Jovicich J Salat D van der Kouwe A Quinn B Czanner S Reliability of MRI-derived measurements of human cerebral cortical thickness: the effects of field strength, scanner upgrade and manufacturer. Neuroimage (2006) 32:180–94. 10.1016/j.neuroimage.2006.02.051 16651008

[50] Varol E Sotiras A Davatzikos C the Alzheimer’s Disease Neuroimaging Initiative HYDRA: revealing heterogeneity of imaging and genetic patterns through a multiple max-margin discriminative analysis framework. Neuroimage (2017) 145:346–64. 10.1016/j.neuroimage.2016.02.041 PMC5408358 26923371

[51] Whitwell JL Dickson DW Murray ME Weigand SD Tosakulwong N Senjem ML Neuroimaging correlates of pathologically defined subtypes of Alzheimer’s disease: a case-control study. Lancet Neurol (2012) 11:868–77. 10.1016/S1474-4422(12)70200-4 PMC3490201 22951070

[52] Adlam A-LR Bozeat S Arnold R Watson P Hodges JR Semantic knowledge in mild cognitive impairment and mild Alzheimer’s Disease. Cortex (2006) 42:675–84. 10.1016/s0010-9452(08)70404-0 16909626

