# Supplementary material for: A Biomarker for Alzheimer’s Disease Based on Patterns of Regional Brain Atrophy
Source: Front Psychiatry. 2020 Jan 14;10:953. doi: 10.3389/fpsyt.2019.00953 (PMC6970941; doi:10.3389/fpsyt.2019.00953)
Supplement: Supplementary file 1 [file DataSheet_1.docx]

## Supplementary Material

### S1 - Magnetic Resonance Imaging Protocols

***Alzheimer's Disease Neuroimaging Initiative (ADNI:***Magnetization prepared rapid gradient-echo (MPRAGE) images were taken with multiple scanners with the following set of parameters: field strength=1.5T, orientation=saggital plane, TR=2300-2400ms or 3000ms, TI=1000ms, flip angle=8°, slice thickness=1.2mm, and resolution 1.25mm x 1.25mm (Jack et al., 2008).

***Open Access Series of Imaging Studies (OASIS):***T1-weighted MPRAGE images were taken with a Siemens Visio scanner with the following set of parameters: field strength=1.5T, orientation=saggital plane, TR=9.7ms, TE=4ms, flip angle=10°, slice thickness=1.25mm, and resolution 1.25mm x 1.25mm.

***Study of Health in Pomerania (SHIP):***

Structural MR images from N=2154 participants of SHIP-Trend screening sample were considered in this study. T1-weighted MPRAGE images of the head were obtained using a Siemens Magnetom Avanto scanner with the following set of parameters: field strength=1.5T, orientation=axial plane, TR=1900 ms, TE=3.37ms, flip angle 15°, slice thickness=1mm, and resolution 1mm x 1mm (Hegenscheid et al., 2009).

### S2 - List of Brain Regions

- - 1. 169 out of 172 brain regions of FreeSurfer’s standard reconstruction were used for training the AD classifier.

***Cortical thickness:***

ctx-lh-bankssts, ctx-lh-caudalanteriorcingulate, ctx-lh-caudalmiddlefrontal, ctx-lh-cuneus, ctx-lh-entorhinal, ctx-lh-fusiform, ctx-lh-inferiorparietal, ctx-lh-inferiortemporal, ctx-lh-isthmuscingulate, ctx-lh-lateraloccipital, ctx-lh-lateralorbitofrontal, ctx-lh-lingual, ctx-lh-medialorbitofrontal, ctx-lh-middletemporal, ctx-lh-parahippocampal, ctx-lh-paracentral, ctx-lh-parsopercularis, ctx-lh-parsorbitalis, ctx-lh-parstriangularis, ctx-lh-pericalcarine, ctx-lh-postcentral, ctx-lh-posteriorcingulate, ctx-lh-precentral, ctx-lh-precuneus, ctx-lh-rostralanteriorcingulate, ctx-lh-rostralmiddlefrontal, ctx-lh-superiorfrontal, ctx-lh-superiorparietal, ctx-lh-superiortemporal, ctx-lh-supramarginal, ctx-lh-frontalpole, ctx-lh-temporalpole, ctx-lh-transversetemporal, ctx-lh-insula, ctx-rh-bankssts, ctx-rh-caudalanteriorcingulate, ctx-rh-caudalmiddlefrontal, ctx-rh-cuneus, ctx-rh-entorhinal, ctx-rh-fusiform, ctx-rh-inferiorparietal, ctx-rh-inferiortemporal, ctx-rh-isthmuscingulate, ctx-rh-lateraloccipital, ctx-rh-lateralorbitofrontal, ctx-rh-lingual, ctx-rh-medialorbitofrontal, ctx-rh-middletemporal, ctx-rh-parahippocampal, ctx-rh-paracentral, ctx-rh-parsopercularis, ctx-rh-parsorbitalis, ctx-rh-parstriangularis, ctx-rh-pericalcarine, ctx-rh-postcentral, ctx-rh-posteriorcingulate, ctx-rh-precentral, ctx-rh-precuneus, ctx-rh-rostralanteriorcingulate, ctx-rh-rostralmiddlefrontal, ctx-rh-superiorfrontal, ctx-rh-superiorparietal, ctx-rh-superiortemporal, ctx-rh-supramarginal, ctx-rh-frontalpole, ctx-rh-temporalpole, ctx-rh-transversetemporal, ctx-rh-insula

***Subcortical gray matter volumes:***
Left-Cerebellum-Cortex, Left-Thalamus-Proper, Left-Caudate, Left-Putamen, Left-Pallidum, Left-Hippocampus, Left-Amygdala, Left-Accumbens-area, Left-VentralDC, Right-Cerebellum-Cortex, Right-Thalamus-Proper, Right-Caudate, Right-Putamen, Right-Pallidum, Right-Hippocampus, Right-Amygdala, Right-Accumbens-area, Right-VentralDC

***Ventricular system volumes:***
Left-Lateral-Ventricle, Left-Inf-Lat-Vent, 3rd-Ventricle, 4th-Ventricle, Left-choroid-plexus, Right-Lateral-Ventricle, Right-Inf-Lat-Vent, Right-choroid-plexus

***Cortical white matter volumes:***
wm-lh-bankssts, wm-lh-caudalanteriorcingulate, wm-lh-caudalmiddlefrontal, wm-lh-cuneus, wm-lh-entorhinal, wm-lh-fusiform, wm-lh-inferiorparietal, wm-lh-inferiortemporal, wm-lh-isthmuscingulate, wm-lh-lateraloccipital, wm-lh-lateralorbitofrontal, wm-lh-lingual, wm-lh-medialorbitofrontal, wm-lh-middletemporal, wm-lh-parahippocampal, wm-lh-paracentral, wm-lh-parsopercularis, wm-lh-parsorbitalis, wm-lh-parstriangularis, wm-lh-pericalcarine, wm-lh-postcentral, wm-lh-posteriorcingulate, wm-lh-precentral, wm-lh-precuneus, wm-lh-rostralanteriorcingulate, wm-lh-rostralmiddlefrontal, wm-lh-superiorfrontal, wm-lh-superiorparietal, wm-lh-superiortemporal, wm-lh-supramarginal, wm-lh-frontalpole, wm-lh-temporalpole, wm-lh-transversetemporal, wm-lh-insula, wm-rh-bankssts, wm-rh-caudalanteriorcingulate, wm-rh-caudalmiddlefrontal, wm-rh-cuneus, wm-rh-entorhinal, wm-rh-fusiform, wm-rh-inferiorparietal, wm-rh-inferiortemporal, wm-rh-isthmuscingulate, wm-rh-lateraloccipital, wm-rh-lateralorbitofrontal, wm-rh-lingual, wm-rh-medialorbitofrontal, wm-rh-middletemporal, wm-rh-parahippocampal, wm-rh-paracentral, wm-rh-parsopercularis, wm-rh-parsorbitalis, wm-rh-parstriangularis, wm-rh-pericalcarine, wm-rh-postcentral, wm-rh-posteriorcingulate, wm-rh-precentral, wm-rh-precuneus, wm-rh-rostralanteriorcingulate, wm-rh-rostralmiddlefrontal, wm-rh-superiorfrontal, wm-rh-superiorparietal, wm-rh-superiortemporal, wm-rh-supramarginal, wm-rh-frontalpole, wm-rh-temporalpole, wm-rh-transversetemporal, wm-rh-insula

***Subcortical white matter volumes:***
Left-Cerebellum-White-Matter, Right-Cerebellum-White-Matter, CC_Posterior, CC_Mid_Posterior, CC_Central, CC_Mid_Anterior, CC_Anterior

### S3 - Tuning Curve


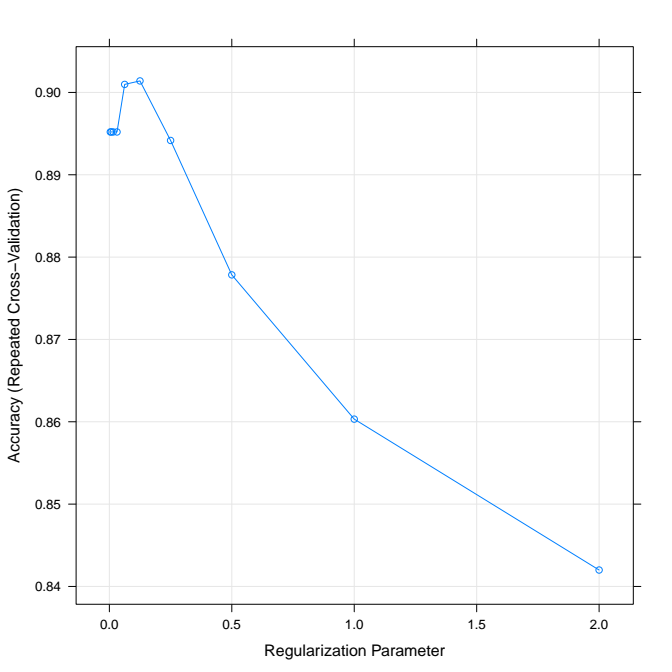


Figure 1 The optimal penalization parameter λ=0.125 was determined by 20-fold cross validation with 20 repetitions.

### S4 – AD Model Coefficients

### i region βi

### 1 Left-Hippocampus 0.3460

### 2 Right-Hippocampus 0.2560

### 3 ctx-rh-entorhinal 0.2375

### 4 ctx-lh-entorhinal 0.2251

### 5 Left-Amygdala 0.2177

### 6 ctx-lh-middletemporal 0.2072

### 7 ctx-lh-bankssts 0.1851

### 8 ctx-lh-parahippocampal 0.1658

### 9 Right-Amygdala 0.1615

### 10 ctx-rh-inferiorparietal 0.1574

### 11 ctx-rh-paracentral -0.1547

### 12 Left-Inf-Lat-Vent -0.1531

### 13 ctx-rh-transversetemporal -0.1518

### 14 ctx-rh-middletemporal 0.1396

### 15 wm-rh-paracentral -0.1391

### 16 ctx-lh-caudalmiddlefrontal 0.1390

### 17 ctx-lh-paracentral -0.1381

### 18 wm-rh-parahippocampal 0.1372

### 19 ctx-lh-inferiortemporal 0.1366

### 20 ctx-lh-rostralmiddlefrontal 0.1326

### 21 CC_Mid_Posterior 0.1316

### 22 Right-Cerebellum-White-Matter -0.1260

### 23 wm-lh-lateraloccipital -0.1252

### 24 wm-lh-parahippocampal 0.1234

### 25 ctx-lh-inferiorparietal 0.1214

### 26 wm-lh-entorhinal 0.1188

### 27 wm-lh-inferiorparietal 0.1124

### 28 wm-lh-paracentral -0.1096

### 29 Left-Putamen -0.0998

### 30 ctx-lh-parsopercularis -0.0989

### 31 ctx-lh-medialorbitofrontal 0.0965

### 32 wm-rh-pericalcarine -0.0938

### 33 ctx-rh-parsopercularis -0.0932

### 34 ctx-rh-lateralorbitofrontal -0.0926

### 35 ctx-lh-rostralanteriorcingulate 0.0911

### 36 ctx-rh-caudalmiddlefrontal 0.0898

### 37 ctx-rh-rostralmiddlefrontal 0.0874

### 38 ctx-rh-inferiortemporal 0.0847

### 39 Right-Inf-Lat-Vent -0.0824

### 40 wm-lh-parsorbitalis -0.0809

### 41 ctx-rh-parahippocampal 0.0791

### 42 ctx-lh-cuneus -0.0789

### 43 wm-lh-isthmuscingulate 0.0768

### 44 wm-lh-pericalcarine -0.0755

### 45 4th-Ventricle 0.0737

### 46 Right-Accumbens-area -0.0731

### 47 ctx-rh-isthmuscingulate 0.0726

### 48 wm-rh-caudalanteriorcingulate -0.0725

### 49 ctx-rh-bankssts 0.0718

### 50 wm-rh-medialorbitofrontal -0.0710

### 51 ctx-lh-fusiform 0.0708

### 52 ctx-lh-caudalanteriorcingulate -0.0685

### 53 wm-rh-isthmuscingulate 0.0677

### 54 wm-rh-precuneus -0.0675

### 55 ctx-rh-precuneus 0.0673

### 56 wm-lh-superiorfrontal -0.0665

### 57 ctx-rh-caudalanteriorcingulate -0.0657

### 58 wm-rh-entorhinal 0.0653

### 59 ctx-rh-medialorbitofrontal 0.0651

### 60 Left-Accumbens-area -0.0646

### 61 wm-rh-bankssts 0.0645

### 62 wm-rh-cuneus -0.0643

### 63 ctx-lh-posteriorcingulate -0.0628

### 64 ctx-lh-transversetemporal -0.0627

### 65 wm-rh-supramarginal -0.0626

### 66 ctx-lh-isthmuscingulate 0.0624

### 67 ctx-rh-cuneus -0.0613

### 68 ctx-rh-insula 0.0612

### 69 wm-rh-transversetemporal -0.0599

### 70 wm-rh-rostralmiddlefrontal 0.0586

### 71 wm-rh-lingual -0.0576

### 72 ctx-lh-precuneus 0.0551

### 73 wm-rh-rostralanteriorcingulate -0.0547

### 74 wm-rh-parstriangularis -0.0537

### 75 CC_Anterior -0.0536

### 76 ctx-lh-insula -0.0528

### 77 wm-lh-caudalmiddlefrontal 0.0525

### 78 Left-Lateral-Ventricle -0.0524

### 79 wm-lh-rostralanteriorcingulate -0.0523

### 80 wm-rh-lateralorbitofrontal 0.0522

### 81 Left-choroid-plexus 0.0517

### 82 3rd-Ventricle 0.0515

### 83 ctx-rh-precentral -0.0495

### 84 wm-lh-frontalpole -0.0488

### 85 wm-rh-middletemporal 0.0482

### 86 Left-Pallidum 0.0470

### 87 ctx-rh-postcentral -0.0468

### 88 Right-Putamen -0.0457

### 89 ctx-lh-supramarginal 0.0452

### 90 ctx-rh-superiorfrontal 0.0451

### 91 ctx-rh-superiortemporal 0.0449

### 92 ctx-rh-fusiform 0.0448

### 93 wm-lh-precuneus -0.0445

### 94 ctx-lh-pericalcarine -0.0444

### 95 wm-lh-lateralorbitofrontal 0.0421

### 96 wm-lh-transversetemporal 0.0416

### 97 wm-lh-fusiform -0.0398

### 98 wm-rh-superiortemporal 0.0391

### 99 wm-rh-inferiortemporal 0.0390

### 100 wm-rh-fusiform 0.0390

### 101 wm-lh-lingual -0.0386

### 102 Left-Cerebellum-White-Matter -0.0383

### 103 ctx-rh-parsorbitalis 0.0381

### 104 ctx-lh-precentral -0.0375

### 105 wm-lh-cuneus -0.0358

### 106 wm-lh-caudalanteriorcingulate 0.0349

### 107 wm-lh-precentral -0.0340

### 108 ctx-lh-frontalpole -0.0335

### 109 wm-rh-postcentral -0.0332

### 110 ctx-lh-lingual -0.0331

### 111 Right-Lateral-Ventricle -0.0330

### 112 ctx-lh-lateraloccipital -0.0330

### 113 ctx-rh-rostralanteriorcingulate -0.0321

### 114 Left-Thalamus-Proper 0.0320

### 115 wm-lh-supramarginal 0.0318

### 116 ctx-rh-supramarginal 0.0306

### 117 Left-Caudate -0.0300

### 118 ctx-lh-lateralorbitofrontal -0.0291

### 119 Right-Caudate 0.0289

### 120 wm-lh-inferiortemporal 0.0282

### 121 ctx-rh-lingual -0.0280

### 122 wm-rh-superiorfrontal -0.0279

### 123 wm-lh-insula -0.0271

### 124 wm-rh-superiorparietal 0.0252

### 125 ctx-lh-parstriangularis -0.0251

### 126 ctx-rh-frontalpole -0.0250

### 127 CC_Posterior 0.0247

### 128 ctx-rh-temporalpole 0.0243

### 129 ctx-lh-parsorbitalis -0.0242

### 130 Right-Thalamus-Proper 0.0240

### 131 Right-choroid-plexus 0.0236

### 132 ctx-lh-postcentral -0.0228

### 133 wm-lh-temporalpole -0.0216

### 134 wm-rh-inferiorparietal 0.0216

### 135 ctx-rh-posteriorcingulate -0.0213

### 136 Left-Cerebellum-Cortex 0.0213

### 137 ctx-lh-temporalpole -0.0200

### 138 wm-rh-caudalmiddlefrontal 0.0189

### 139 ctx-rh-superiorparietal -0.0182

### 140 ctx-lh-superiorparietal 0.0180

### 141 wm-lh-parstriangularis 0.0176

### 142 Right-Cerebellum-Cortex 0.0173

### 143 ctx-lh-superiortemporal 0.0171

### 144 wm-lh-rostralmiddlefrontal 0.0166

### 145 wm-lh-medialorbitofrontal -0.0166

### 146 wm-rh-frontalpole -0.0162

### 147 wm-lh-superiorparietal 0.0155

### 148 wm-lh-posteriorcingulate 0.0155

### 149 wm-lh-superiortemporal 0.0143

### 150 Left-VentralDC 0.0130

### 151 Right-VentralDC 0.0123

### 152 wm-lh-middletemporal 0.0110

### 153 ctx-lh-superiorfrontal 0.0108

### 154 wm-lh-bankssts -0.0106

### 155 CC_Mid_Anterior 0.0105

### 156 CC_Central 0.0100

### 157 wm-lh-postcentral -0.0097

### 158 ctx-rh-pericalcarine 0.0094

### 159 wm-rh-insula -0.0094

### 160 wm-rh-precentral -0.0081

### 161 wm-rh-parsopercularis 0.0078

### 162 Right-Pallidum 0.0063

### 163 wm-lh-parsopercularis -0.0043

### 164 ctx-rh-lateraloccipital 0.0039

### 165 wm-rh-lateraloccipital 0.0027

### 166 wm-rh-parsorbitalis 0.0018

### 167 ctx-rh-parstriangularis -0.0017

### 168 wm-rh-posteriorcingulate 0.0013

### 169 wm-rh-temporalpole 0.0000
